# Microwave-assisted xylanase reaction: impact in the production of prebiotic xylooligosaccharides†

**DOI:** 10.1039/d1ra00449b

**Published:** 2021-03-23

**Authors:** Hugo Mobarec, Rodrigo Villagomez, Eva Nordberg Karlsson, Javier A. Linares-Pastén

**Affiliations:** Lund University, Division of Biotechnology, Department of Chemistry Lund Sweden javier.linares_pasten@biotek.lu.se; Lund University, Centre for Analysis and Synthesis Lund Sweden; Technical University of Denmark, Department of Chemical and Biochemical Engineering Copenhagen Denmark

## Abstract

The enzymatic production of prebiotic xylooligosaccharides (XOS) has become an attractive way to valorise lignocellulosic biomass. However, despite numerous xylanases reported for potential use in the production of XOS, most of the family GH10 also produce xylose. This monosaccharide can negatively affect the selectivity to stimulate the growth of intestinal microorganisms beneficial to human health. In this work, thermostable alkali-tolerant xylanase (*Bh*Xyn10A) from *Bacillus halodurans* S7 has been used to produce XOS under conventional convective heat transfer and microwave radiation. The microwave-assisted reaction markedly decreases the xylose content in the hydrolysates and significantly increases the yield of XOS, compared to conventional heating. Molecular dynamics simulations of *Bh*Xyn10A have shown increased fluctuations of the amino acids of the aglycone subsites suggesting that these subsites can determine the production of xylose. Thus, microwave heating could affect the amino acid fluctuations in the aglycone subsites reducing the xylose formation. These findings open up new avenues in enzyme technology for the production of XOS.

## Abstract 2

The microwave-assisted reaction markedly decreases the xylose content in the hydrolysates and significantly increases the yield of XOS, compared to conventional heating.

## Graphical abstract

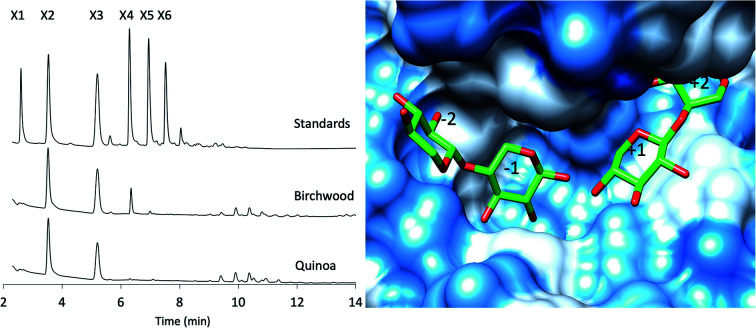

## 1. Introduction

Lignocellulosic biomass has been gaining increased attention because of its potential as a raw material for the biochemical industry from a biorefinery perspective. After a pre-treatment stage, lignocellulosic biomass can be fractionated into its three main components – cellulose, hemicellulose, and lignin – from which each one can be subsequently processed separately for manufacturing a wide variety of products. For instance, hemicelluloses can be transformed into several molecules such as xylitol, erythritol, ferulic acid, furfural, ethanol, lactic acid, and xylooligosaccharides (XOS).^1^

Particular interest has been put into the production of XOS, they can selectively stimulate the growth of probiotic bifidogenic and lactic acid bacteria residing in the human gut.^2^ Nonetheless, it has also been found that it is mostly XOS with a degree of polymerization (DP) from 2 to 4 that selectively induce the growth of beneficial microorganisms in the human digestive tract.^2^ Therefore, the xylose (DP1) content should be minimized to enhance the quality of the prebiotic mixture.

The hydrolysis of xylan-rich hemicelluloses into XOS can be attained by enzymatic conversion. Xylanases can be either *endo*-acting (EC. 3.2.1.8) or *exo*-acting enzymes (EC. 3.2.1.156 and 3.2.1.37). Since *exo*-acting xylanases release xylose monomers (DP1), only *endo*-acting xylanases are of interest for XOS production.^3,4^

It has been found that the composition profile of the products after the xylanase-mediated hydrolysis significantly differs dependent on the enzyme used and the reaction conditions, such as time, temperature, and substrate.^5^ Henceforth, optimizing both the proportion of XOS with oligomers (DP2–4) in the mixture and the hydrolysis yield will be key factors for designing an effective production process. For this purpose, it has been suggested that microwave radiation might have a synergic effect in combination with enzyme catalysis, favouring both the yield and the product selectivity.^6^ In addition, microwave radiation has been proposed as a green technology with application in biomass extraction.^7^

Thermostable enzymes are attractive biocatalysts in a variety of biorefinery processes,^5^ due to their stability and capacity to perform reactions in high temperatures. At these conditions, polymeric substrates, such as xylan, are more soluble, making the reaction mixture lower viscous, increasing the yield and the efficiency of the process. Besides, these enzymes can be coupled to microwave reactors due to their thermostability, allowing the development of green-chemistry processes for the manufacture of novel products.

The objective of this work was to investigate the effect of microwave-assisted heating in the enzymatic production of XOS. Three variants of a thermostable alkali-tolerant xylanase from *Alkalihalobacillus halodurans* (*Bacillus halodurans* S7)^8^ were studied: (1) *Bh*Xyn10A, is the wild type (*wt*) form, (2) *Bh*Xyn10K80R, is a mutant that has shown a higher activity than the *wt* under conventional conditions,^1^ (both with a C-terminal histidine tag) and (3) *Bh*HXyn10A, is a variant containing an N-terminal addition, including the histidine tag.^9^ Xylan from birchwood and quinoa were used as substrates. Additionally, ligand/enzyme docking, and molecular dynamics simulations were performed to study the structural implications in the production of XOS and xylose.

## 2. Material and methods

### 2.1. Chemicals

Xylan from birchwood, beechwood, and Larchwood, were purchased from Sigma Aldrich (Saint Louis, Missouri). Xylan from quinoa (*Chenopodium quinoa*) stalks was extracted as described in our previous work.^10^ Analytical grade xylose, xylobiose, xylotriose, xylotetraose, xylopentaose, and xylohexaose were obtained from Megazyme (Wicklow, Ireland).

### 2.2. Enzymes: production and purification

#### 2.2.1. Production

*Bh*Xyn10A, *Bh*Xyn10K80R, and *Bh*HXyn10A enzymes encoded by a synthetic gene (GeneBank accession number: MW311490) were produced in *Escherichia coli* BL21(DE3) in 2.5 L bioreactors as described in our previous work.^11^ Pre-inoculums were prepared in 100 mL of mAT medium^12^ with 10 g L^−1^ glucose as sole carbon source. *E. coli* strains harboring plasmids pET21::*Bh*Xyn10A, and pET21::*Bh*Xyn10AK80R were inoculated in culture media supplemented with 100 μg mL^−1^ ampicillin, while the medium for the strain *E. coli* 21(DE3) pET28::*Bh*HXyn10A was supplemented with 34 μg mL^−1^ kanamycin. All pre-inoculums were grown for 12 hours in shake flasks at 30 °C. Reactors containing 2.4 L mAT medium and set up at 37 °C and 40% O_2_ saturation, were inculcated with 100 mL of inoculums previously described. Recombinant protein expression was induced with 1 mM isopropyl-β-d-thiogalactopyranoside (IPTG) when the optical density of the cultivations reached 3 at 600 nm. The induction period was for 2 hours. Cell pellets were harvested by centrifugation (4500*g*), 10 min, for protein purification, while the supernatants were discarded.

#### 2.2.2. Purification

Cell pellets were resuspended in a binding buffer consisting of 20 mM Tris–HCl, 0.5 M NaCl, pH = 7.4; and lysed by ultrasonication in intervals of 10 × 10 min. Next, lysates were centrifuged at 5500*g* for 20 min, pellets were discarded, and supernatants were used for the protein purifications by immobilized metal ion affinity chromatography (IMAC). An ÄKTA prime system (Amersham Biosciences, Sweden) with a 5 mL FF HisTrap nickel column (GE Health Care, Germany) was used. The method was: column equilibration with 5 volumes of binding buffer, injection of samples (supernatants) through the system, washing with binding buffer until reaching the equilibration-baseline absorbance, and elution with a linear gradient of buffer 20 mM Tris–HCl, 0.5 M NaCl, 0.5 M imidazole, pH 7.4. Purity and concentration of the purified proteins were analysed by SDS-PAGE and spectrophotometry respectively.

### 2.3. Enzymatic reactions

#### 2.3.1. Conventional heating reactions

Four types of xylan, from birchwood, beechwood, Larchwood, and quinoa stalks, were used as substrates for the three enzyme variants (*Bh*Xyn10A, *Bh*Xyn10K80R, and *Bh*HXyn10A) of the alkali-tolerant *Bacillus halodurans* S7 endoxylanase, giving in total twelve different reactions. Every reaction was performed in triplicate, as well as the corresponding controls without enzyme. The reaction volume was 200 μL, containing, 1% xylan, in 100 mM glycine-NaOH buffer pH 9, and 6.67 mg L^−1^ of the enzyme. The reactions were incubated at 62 °C for 15 h. The reactions were started by adding the enzyme and stopped by heating at 100 °C for 10 min.

#### 2.3.2. Microwave assisted reactions

Xylan from birchwood and quinoa stalks were used as substrates. Every reaction mix was prepared up to a final volume of 6 mL, in 20 mL microwave vials provided with a magnet for mixing. The components were 1% xylan, 100 mM glycine-NaOH buffer pH 9, and 6.67 mg L^−1^ of the enzyme. The reactions were heated in an Initiator+ Microwave Synthesizer (Biotage, Sweden) reactor. The reaction mixtures were irradiated at 62 °C for 0 to 30 min, the time was counted after this temperature was reached. The power input was 40 W, with the microwave power delivery system ranging from 0 to 40 W. The reactions were stopped deactivating the enzyme by heating for 10 min at 100 °C.

### 2.4. Temperature profiles

Xylanase *Bh*Xyn10A activity was determined in a range of temperatures from 40 to 85 °C, both in conventional as well as microwave heated reactions. The reaction mixtures were prepared up to a final volume of 6 mL, containing 1% birchwood xylan, 50 mM glycine buffer pH 9, and 6.67 mg L^−1^ of the enzyme. Activities were quantified after 10 min of incubation by DNS method for reducing end sugars such is described below (Section 2.5.2.).

### 2.5. Analytical methods

#### 2.5.1. Protein analysis

Recombinant protein expression level and protein content in purified fractions were analysed by SDS-PAGE. The concentration of the purified enzymes was determined in a NanoDrop instrument (Thermo Fisher Scientific, United States of America) and corroborated by spectrophotometry at a wavelength of 280 nm.

#### 2.5.2. Enzyme activity analysis

Products of the enzymatic reactions, after incubations using conventional heating and microwave assisted heating, were analysed by a colorimetric assay, using 3,5-dinitrosalicylic acid (DNS) for quantifying reducing sugars.^13^ The DNS solution contained a 1 : 1 : 1 : 1 volumetric mixture of 1% DNS, 40% Rochelle salt, 0.2% phenol, 0.5% potassium disulfide, and all these components were mixed in 1.5% sodium hydroxide. A volume of 400 μL of the hydrolysate was mixed with 600 μL of DNS solution and heated to 100 °C for 10 minutes. Subsequently, the obtained coloured solution was diluted 10 times and then transferred to a microplate well for its quantification at 540 nm in a microplate spectrophotometer (Thermo Scientific™, Multiskan™ GO). The actual concentrations were calculated based on a calibration curve using xylose as standard in a concentration range of 0 to 2 μmol.^1^

#### 2.5.3. Oligosaccharide analysis

Oligosaccharides produced both in conventionally heated and microwave-assisted reactions were analysed by high-performance anion-exchange chromatography with pulsed amperometric detection (HPAEC-PAD). A Dionex chromatography system with a PA-100 column (Thermo Fisher Scientific, United States of America) was used, such as described in our previous work.^9^

### 2.6. Computational studies

#### 2.6.1. Construction of the complex enzyme/ligands

The following model of a complex between the enzyme and ligands (products of hydrolysis) was built: *Bh*Xyn10AH/β-d-Xylp-(1-4)-β-d-Xylp-(1-4)-β-d-Xylp/β-d-Xylp-(1-4)-β-d-Xylp. The atomic coordinates of the ligands were transferred from the crystallographic structure of the complex *Streptomyces olivaceoviridis* E-86 (PDB: 1ISX) to the active site of the crystallographic structure of the apoenzyme *Bh*Xyn10AH (PDB: 2UWF), by superposition of both structures using CHIMERA v1.14.^14^ Thereafter, the reducing-end xylose of the xylotriose located in the aglycone side was removed. The obtained complex was energetically minimized using YASARA v18.4.24 ^15^ with the AMBER14 force field.^16^

#### 2.6.2. Molecular dynamic simulations

The minimized complex was subjected to molecular dynamics simulations with explicit molecules of water as a solvent. All calculations were performed in YASARA v18.4.24,^15^ using AMBER14 force field.^16^ A cubic simulation cell, 20 Å larger than the complex, with periodic boundary conditions, was filled with TIP3P water molecules and counter ions.^17^ The distance for Van der Waals interactions was set in a medium range of 8 Å, and the long-range Coulomb forces were calculated using the particle-mesh Ewald algorithm.^18^ The temperature control was through the Berendsen Thermostat.^19^ The simulated conditions were: 0.9% NaCl, pH 9, 0.982 g mL^−1^ solvent density, and 335 K during 50 ns, saving snapshots every 100 ps. Root-mean-square deviations (RMSD) of atomic coordinates, root-mean-square fluctuations (RMSF), and enzyme/ligand interactions were analysed.

## 3. Results and discussion

### 3.1. Enzymes production

Three variants (*Bh*Xyn10A, *Bh*Xyn10K80R, and *Bh*HXyn10A) of the 1,4-β-*endo*-xylanase from *Bacillus halodurans* S7 were successfully produced in *E. coli* BL21(DE3) harbouring synthetic genes. *Bh*Xyn10A is the wild type form with a histidine tag in the C-terminus; *Bh*Xyn10-K80R has an arginine residue instead of lysine in position 80. The form *Bh*HXyn10A has an additional tail of 16 amino acids and a histidine tag in the N-terminus. The recombinant strains were cultivated in a batch reactor (2.5 L) using the synthetic medium mAT^12^ with 10 g L^−1^ of glucose (S) as the sole carbon source. High productivity (Table 1) of soluble recombinant protein was reached after 5 h of cultivation, during which the last 2 h were the production phase initiated with the addition of the inducer (IPTG). All three enzyme variants were purified by IMAC, prior to use for XOS production.

**Table 1:** Yield coefficients, *Y*_P/S_ (mass of recombinant protein/mass of substrate), *Y*_P/X_ (mass of recombinant protein/biomass), and volumetric productivity (*Q*_P_) of recombinant variants of 1,4-β-*endo*-xylanase from *Bacillus halodurans* S7 in *E. coli* BL21(DE3) in batch cultivations

Productivity	*Bh*HXyn10A	*Bh*Xyn10AH	*Bh*Xyn10K80R
*Y*_P/S_ (g g^−1^)	0.04	0.13	0.04
*Y*_P/X_ (g g^−1^)	0.06	0.26	0.12
*Q*_(P)_ (g L^−1^ h^−1^)	0.04	0.15	0.05

### 3.2. Production of XOS in conventionally heated reactions

**Fig. 1:**
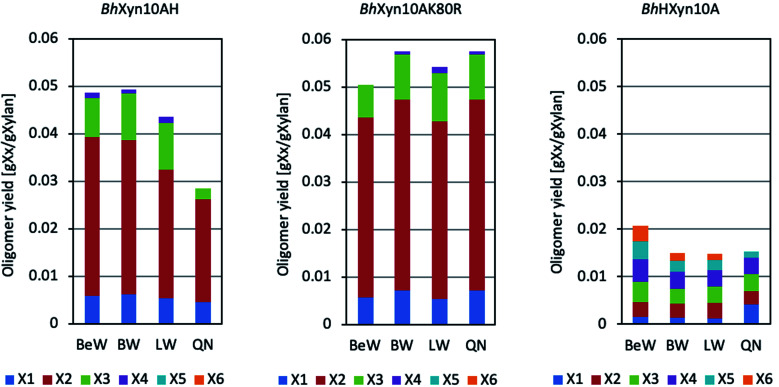
Product profiles of the enzymatic conversion of different xylans to xylose and XOS under conventional heating. Substrates were: BeW (beechwood xylan), BW (birchwood xylan), LW (Larchwood xylan), and QN (quinoa xylan). Analyzed products were X1 (xylose), X2 (xylobiose), X3 (xylotriose), X4 (xylotetraose), X5 (xylopentaose) and X6 (xylohexaose). Variants *Bh*Xyn10AH, *Bh*Xyn10K80R and *Bh*HXyn10A of *Bacillus halodurans* 1,4-β-endoxylanase were used.

Xylans from birchwood, beechwood, larchwood, and quinoa stalks were used as substrates for each variant of the 1,4-β-endoxylanase from *B. halodurans* (Fig. 1). *Bh*Xyn10A and *Bh*Xyn10K80R have shown similar product profiles on all the substrates used, with the main products being xylobiose (X2), xylotriose (X3), and xylose (X). However, the mutant K80R yielded a slightly higher amount of the products mentioned. This result is even more pronounced when the substrate is xylan from quinoa (Fig. 1). These results are consistent with the higher activity of the mutant K80R relative to the wild type, as reported previously,^1^ which also resulted in a slightly higher yield of XOS from quinoa xylan.

On the other hand, the variant *Bh*HXyn10A produced a lower total amount of XOS (lower than 20 mg XOS per 1 g of xylan). However, interestingly, its product profile was different from the other two variants, since relatively long XOS, such as xylotetraose (X4), xylopentaose (X5), and xylohexaose (X6), were produced in similar amounts as X2 and X3 (Fig. 1). This type of products profile was first obtained after hydrolysis of quinoa xylan, and later in this work also after hydrolysis of beechwood, birchwood, and larchwood xylans. Thus, the elongation with the N-terminal tail in the variant *Bh*HXyn10A affected significantly both the yield and the product profile.

The monosaccharide xylose was produced in all of the reactions, independent of the substrate type or endoxylanase variant. However, xylose is an undesirable co-product since it does not selectively promote probiotic growth, as XOS do. Endoxylanases with a low rate of xylose production are thus preferred in the reaction development for prebiotic XOS production. Microwave-assisted reactions have been shown to increase the yield and specificity of reactions catalysed by both enzymatic and non-enzymatic catalysts.^20^ Therefore, we have here hypothesized that the microwave-assisted endoxylanase reaction can reduce the co-production of xylose and enhance the production of XOS.

### 3.3. Production of XOS in microwave-assisted reactions

Microwave pre-treatment of xylan has been shown to improve subsequent enzymatic hydrolysis^21^ under conventional heating. However, to the best of our knowledge, the effect of microwave irradiation during the action of the endoxylanase has not been reported prior to this work.

**Fig. 2:**
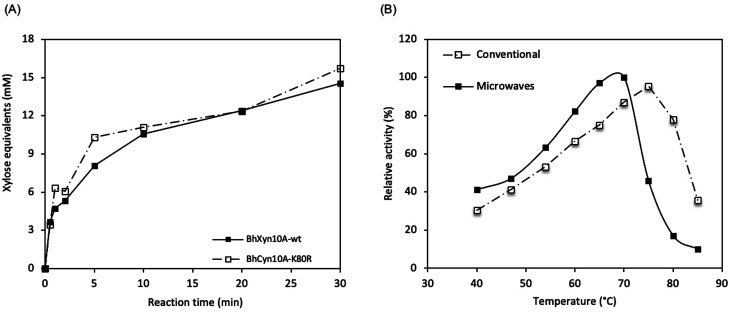
Microwave-assisted enzymatic reactions. (A) Time courses of birchwood xylan hydrolysis catalysed by *Bh*Xyn10A and variant *Bh*Xyn10AK80R. (B) Temperature profiles of the activity of *Bh*Xyn10A both in conventional as well as in microwave heated systems.

Here, it was found that the microwave-assisted enzymatic reaction resulted in significantly higher activity than the conventionally heated enzyme reaction. Initially, the reaction time and optimal temperature for the microwave assisted reactions were determined by quantifying the total reducing end sugars (DNS assay) produced enzymatically from birchwood xylan. The reaction was monitored for 30 min at 62 °C and pH 9. It was seen that at these conditions, even after only some seconds of the reaction, a detectable amount of product was obtained (Fig. 2A). This observation is valid for the two enzymes *Bh*Xyn10A and *Bh*Xyn10AK80R. However, no activity was detected for the variant *Bh*HXyn10A. Since the other two variants appear to be working efficiently, denaturation might not be a likely explanation. It could be that the added tag (16 amino acids including the histidine tag) attached to the N-terminus, under microwave radiation gets oriented in such a way that it interferes with the active site of the enzyme. But further research is needed to confirm this claim.

Based on the tendency of increasing activity with time over the complete time course, as seen in the curves for the two variants *Bh*Xyn10A and *Bh*Xyn10AK80R (Fig. 2A), it could be suggested that the enzymes are not completely deactivated after the 30 min reaction under microwave radiation. This indicates that the enzymatic process is robust at the selected power input and reaction time, and even opens the possibility to increase both parameters, to further increase the yields. Overall, the mutant variant *Bh*Xyn10AK80R performs slightly better than the *wt Bh*Xyn10A, an observation which is consistent with the higher activity of the mutant compared to the *wt* at conventional conditions.^1^

The optimal temperature for *Bh*Xyn10A under microwave-assisted heating is 68 °C while under conventional heating it is higher, 75 °C (Fig. 2B). However, the relative activity is significantly higher under microwave irradiation at temperatures below 70 °C. These results show that the enzyme is less tolerant to high temperatures in the presence of microwaves than in conventional heating.

Microwave-assisted reactions were initially carried out on birchwood xylan to observe the evolution of the profile with time. Only the enzyme *Bh*Xyn10A and *Bh*Xyn10AK80R were taken into account since *Bh*HXyn10A had been determined to be inactive under the studied experimental conditions. The reactions were also performed on quinoa xylan at two time points in the incubation interval used.

**Fig. 3:**
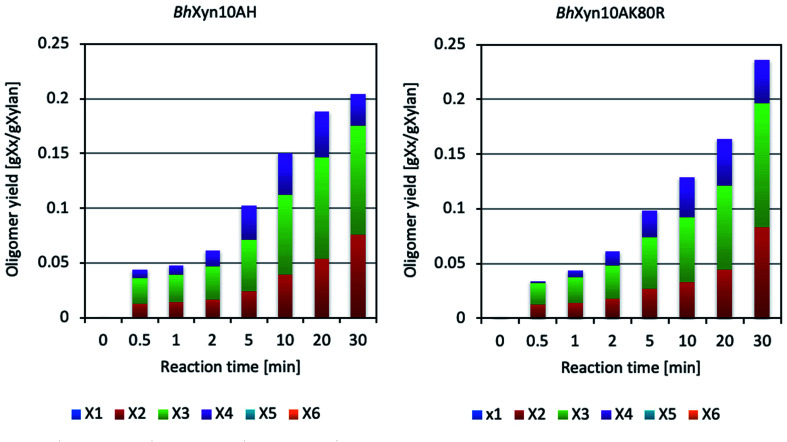
Composition profiles of the hydrolysates obtained by microwave-assisted enzymatic conversion of birchwood xylan. The analyzed oligomers are X1 (xylose), X2 (xylobiose), X3 (xylotriose), X4 (xylotetraose), X5 (xylopentaose) and X6 (xylohexaose).

According to Fig. 1, the enzymatic conversion of xylan to XOS, using the four types of xylans at conventional heating conditions produced a wide range of products, including a considerable proportion of xylose in all cases. Moreover, it can be seen that the variant *Bh*HXyn10A was active at the conventional heating conditions, even though its yield was significantly lower. On the other hand, microwave-assisted conversion of birchwood and quinoa xylans did not produce any detectable amount of xylose at any of the studied time points in the interval (Fig. 3). Also, it is important to note that the controls, which did not contain any enzyme and were only inactivated by microwave heating (100 °C), did not experience any detectable thermal hydrolysis. Therefore, spontaneous release of oligomers at high temperatures can be neglected.

It is interesting to note that despite the marked improvement in the XOS yield obtained by microwave-assisted heating in relation to traditional heating (approximately fivefold as seen when comparing Fig. 1 and 3), the reaction curves constructed based on the DNS assay show that the total concentration of products containing reducing end sugars is very similar in both cases, indicating a profile of longer oligosaccharides in the microwave-assisted reaction. Further studies would be needed to analyse the proportion of xylooligosaccharides longer than six carbons in the obtained hydrolysates, but it should be highlighted that microwave-assisted heating produced a significantly higher yield of XOS with a DP from 2 to 4 (Fig. 3) (instead of DP1-3 in the conventional reaction, Fig. 1), which is an adequate XOs-range to specifically favour the beneficial microbes in the human gut.^2^

### 3.4. Structural studies

**Fig. 4:**
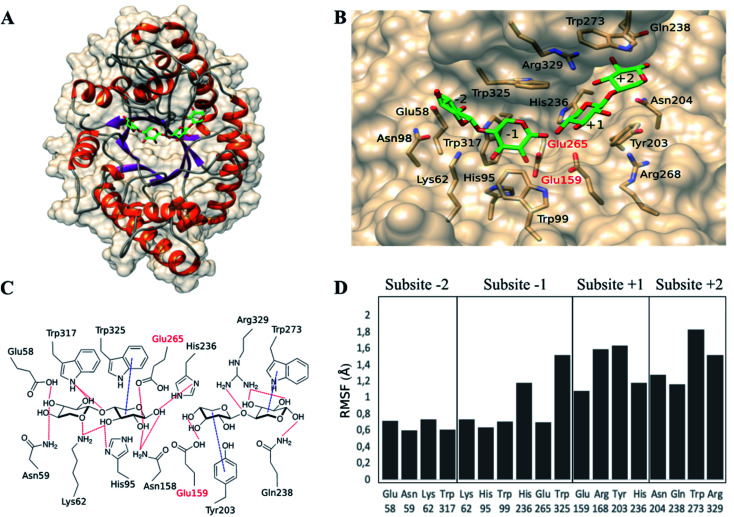
Complex enzyme/ligands *Bh*Xyn10AH/β-d-Xylp-(1-4)-β-d-Xylp-(1-4)-β-d-Xylp/β-d-Xylp-(1-4)-β-d-Xylp, modelled based on the crystallographic structure of the apoenzyme *Bh*Xyn10AH (2UWF). Ligands are represented in green (carbon atoms) and red (oxygen atoms). (A) Overall view of the enzyme/ligands model. (B) Predicted subsites and interactions between the active site amino-acids and ligands. Catalytic glutamates are labelled in red (Glu265 and Glu159). (C) Representation of the predicted interactions between enzyme and ligands. (D) Root-mean-square-fluctuations (RMSF), obtained by molecular dynamic simulations (50 ns), of the amino-acids surrounding every subsite, some of them interact with the ligands.

To find an explanation of the microwave effect on the reduced production of xylose, computational studies of the complex between xylanase and ligands were performed. First, the complex *Bh*Xyn10A/β-d-Xylp-(1-4)-β-d-Xylp-(1-4)-β-d-Xylp/β-d-Xylp-(1-4)-β-d-Xylp was built (Fig. 4A) based on available crystallographic structures. The ligand of the co-crystallized structure of *Streptomyces olivaceoviridis* E-86 (PDB: 1ISX) was after superimposition transferred to the apo 3D-structure of the xylanase from *Bacillus halodurans* (*Bh*Xyn10A) (PDB: 2UWF). The structure of the overall complex was then energetically minimized as described in Section 2.6.

*Bh*Xyn10AH has an α/β-barrel 3D structure as all glycoside hydrolases from family GH10 (www.cazy.org) (Fig. 4A). The catalytic site is a cleft containing at least four recognized subsites (Fig. 4B): −2 and −1 are the glycone subsites and +1 and +2 are the aglycone subsites. The catalytic amino acids, Glu265 and Glu159, were verified by superimposing the modelled ligand-complex *Bh*Xyn10A and the reference structure 1ISX. The enzyme/ligand model also allowed prediction of amino acid/ligand interactions at each subsite (Fig. 4C). Glycone subsites were shown to contain more potential hydrogen bonds than the aglycone subsites, which has also been observed in other xylanases.^22^

Subsequently, the complex was subjected to molecular dynamics simulations to study the fluctuations in the interaction of each amino-acid residue with the ligands (ESI S1 and S2†). The root-mean-square-fluctuations (RMSF) showed that the amino acids in subsites +1 and +2 are more flexible than those located in subsites −2 and −1 (Fig. 4D). Furthermore, the density of hydrogen bonds is higher in the glycone than the aglycone subsites, indicating tighter ligand binding interactions in the glycone subsites. All this suggests, that aglycone subsites are less specific, but can bind either two or one xylose units under conventional conditions, resulting in the production of xylose as co-product. Therefore, according to our experimental results, we hypothesize that microwave radiation may have a stronger effect on the ligand-interactions on aglycone than glycone subsites. It is interesting to notice that aglycone subsites show higher fluctuations than glycone subsites. Thus, it is likely that the microwave-assisted heating reduces the possibility to only bind a single xylose moiety in the aglycone part of the active site, instead requiring interactions of at least two xylose subunits, thereby reducing the xylose formation.

In general, the molecular mechanisms associated with the effects of microwaves in chemical reactions have not yet been fully elucidated. Microwaves are non-ionizing electromagnetic radiation, which is absorbed at the molecular level causing changes in the vibrational energy of the molecules. These motions produce collisions, friction, and the loss of absorbed energy in the form of heat.^20,23^ Therefore, we hypothesise that the residues with grater fluctuations (RMSF) are more sensitive to the microwave effects, as observed in the aglycone subsites of the xylanase studied. Aglycone subsites have shown weaker interactions with the ligand, which can be further weakened by microwave radiation, disfavouring the binding of a xylose moiety in +1 necessary for xylose production. However, larger ligands can allow the production of XOS due to a greater number of potential interactions, especially in subsite +2. More research is, however, needed to provide further insights into the mechanisms of microwave radiation on enzyme activity.

## 4. Conclusion

The microwave-assisted heating reaction markedly decreases the xylose content in the hydrolysates, and significantly increases the yield of XOS, compared to conventional heating reactions. Based on molecular dynamic simulations of *Bh*Xyn10A, we suggest that microwave heating affects the amino acid interactions with the substrate in the aglycone subsites, thereby reducing the xylose formation. The microwave-assisted heating yields a product that is significantly richer in xylooligosaccharides with a degree of polymerization that might selectively induce the growth of beneficial microorganisms in the human gut.

## Author contributions

Conceived and designed experiments: J. A. Linares-Pastén, R. Villagomez. Performed experiments: H. Mobarec, R. Villagomez, J. A. Linares-Pastén. Analyzed data: H. Mobarec, R. Villagomez, J. A. Linares-Pastén, E. Nordberg Karlsson. Contributed materials/analysis tools: J. A. Linares-Pastén, E. Nordberg Karlsson. Wrote the paper: all authors.

## Conflicts of interest

There are no conflicts to declare.

## Supplementary Material

RA-011-D1RA00449B-s001
