# Supplementary material for: Microwave-assisted xylanase reaction: impact in the production of prebiotic xylooligosaccharides
Source: RSC Adv. 2021 Mar 23;11(20):11882–8. doi: 10.1039/d1ra00449b (PMC8696575; doi:10.1039/d1ra00449b)
Supplement: RA-011-D1RA00449B-s001 [file RA-011-D1RA00449B-s001.pdf]

Electronic Supplementary Material (ESI) for RSC Advances.
This journal is © The Royal Society of Chemistry 2021

Supplementary information

Microwave-assisted xylanase reaction: the impact in the production of prebiotic xylooligosaccharides

Hugo Mobarec[1,3], Rodrigo Villagomez[2], Eva Nordberg Karlsson[1], Javier A. Linares-Pastén[1,*]

[1] Lund University, Division of Biotechnology, Department of Chemistry, Lund, Sweden
[2] Lund University, Centre for Analysis and Synthesis, Lund University, Lund, Sweden
[3] Technical University of Denmark, Department of Chemical and Biochemical Engineering, Copenhagen, Denmark

*Corresponding author: J. A. Linares-Pastén
(e-mail: *javier.linares_pasten@biotek.lu.se*)

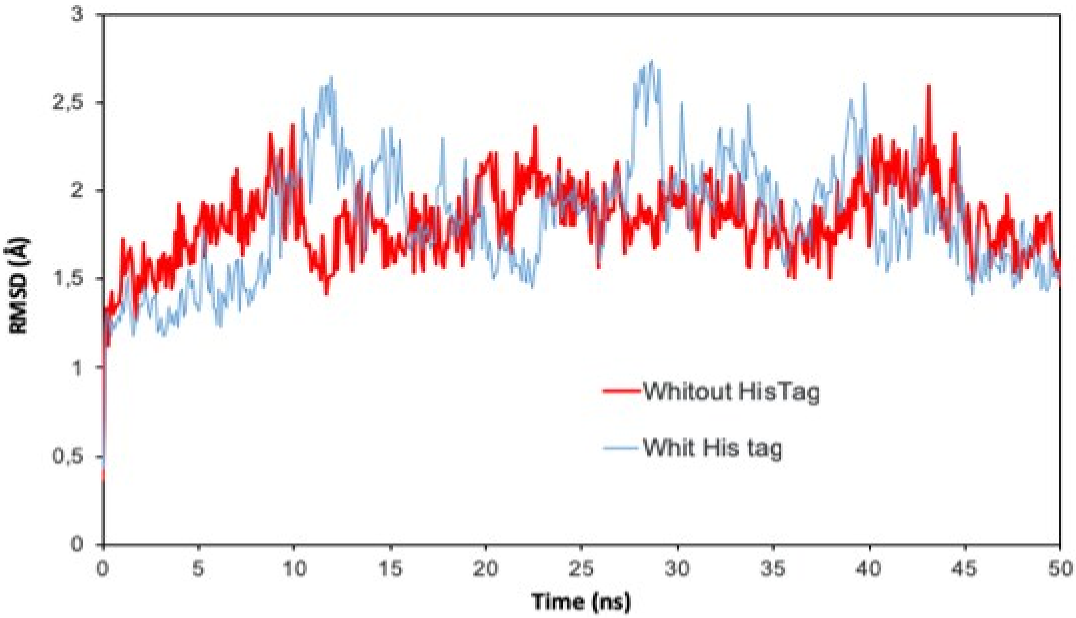

Figure 1S. Root-mean-square-deviations (RMSD) of the complex *Bh*Xyn10AH/ligands with and without 6x C-histidine tag.

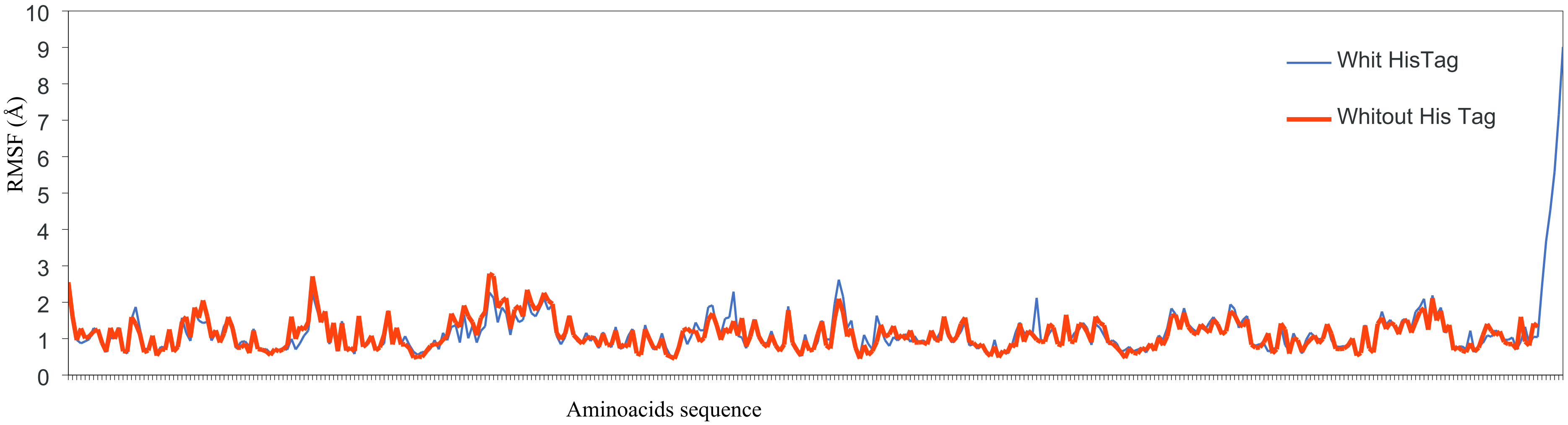

Figure 2S. Root-mean-square-fluctuations (RMSF) of the complex *Bh*Xyn10AH/ligands obtained by molecular dynamic simulations during 50 ns. Comparison of structures with and without 6x C-histidine tag. Notice that the histidine tag is very flexible.
